# Institutional Notes and News

**Published:** 1909-12-11

**Authors:** 


### Institutional Notes and News.

### JUBILEE OF THE NATIONAL HOSPITAL (ALBANY MEMORIAL).

Mr. Burford Rawlings is writing a history of the Institution, which, we understand, will be published early in the coming spring.

### THE NEWARK HOSPITAL.

With regard to the action of the officials in not admitting a woman from Shelton, a special meeting of the Board of Governors has been held, at which the following decisions were arrived at :—That no ground of complaint was substantiated against the management of any member of the hospital staff. That the resident medical officer was perfectly right in the course he adopted, and is fully entitled to the support of the Board. That the resident medical officer and the matron treated Mrs. Lacey with every consideration and kindness, and the Board approve of their action. That the Board very much regret the course adopted by Colonel Vere Wright in addressing his complaint to the newspapers instead of laying it before the Board in the usual way, as coming from a gentleman in his position it is calculated to create a prejudice against the institution and to do it considerable financial injury.

### SHEFFIELD ROYAL HOSPITAL.

At the annual meeting recently the 77th report of the Board of Management stated that the next block of the new building will be completed early in 1910. The new building fund now amounts to £22,753 11s. 9d., including Mr. Edgar Allen's handsome donation of £5,000. This generosity has enabled the Board to consider the question of the entire completion of the hospital, and plans for the final wing in Eldon Street have been tentatively prepared. The sum of £8,000 is still needed to complete the scheme. A special aural department has been created, while Mr. W. Edgar Allen has presented to the electrical department a complete *x*-ray installation with the most modern appliances. The report referred in fitting terms to the death of Dr. Keeling, who was connected with the hospital for 42 years, and stated that as a slight recognition of his valuable services a ward in the new wing would be named the Keeling Ward. The Duke of Norfolk, in moving the adoption of the report, dwelt upon the painful fact that more than £3,000 had had to be taken from capital to balance the accounts for the year. Legacies, he said, made up about two-thirds of the deficit, but he was told that legacies showed a tendency to decline. That was a distressing fact, and, seeing the growth of Sheffield and the work to be done, no source of income could be disregarded. The report having been adopted, the Duke of Norfolk was unanimously re-elected president of the hospital, and the Duchess president of the Samaritan Society. The Lord Mayor, in reply to the resolution of thanks to the Board of Management, referring to himself, said they heard a good deal about the municipalisation of hospitals, but he did not think the time for that was ripe, even if it ever would be. If it did come, the hospitals would lose that individual effort which did so much for their success.

### SUSSEX COUNTY HOSPITAL.

At a special General Court of Governors recently the following resolution was adopted :—"That sanction be given for the acquisition of about five acres of land adjoining the Hospital, the cost of which is being defrayed by two members of the Committee of Management." The consideration of this question has occupied the attention of the Committee for some time past, and there is no doubt as to the advantage which will follow from the acquisition of the land, as it will for all time prevent others from building close to the Hospital, and will ensure a continuance of the magnificent air which the patients have hitherto enjoyed. The difficulty which the Committee had to face was the cost of the land, and it was thought in the present financial condition the Institution was not justified in spending so much money. That difficulty has been removed by the great generosity of two members of the Committee, Messrs. C. Borradaile and C. C. Baily. The new land, which has been obtained from the Marquess of Bristol, lies to the north-east of the Hospital, and extends about 120 feet from the northern boundary and as far as the Bristol Gate on the east. No definite plans for utilising it have been made at present, but great satisfaction is felt that it will be available when required, and that in the meantime no other buildings can be erected on it.

### CARDIFF INFIRMARY.

Mrs. John Nixon, who contributed 10,000 guineas to the building fund, laid the foundation-stone of the new wing on October 21. In 1906 the old out-patient department was found to be quite inadequate, and Colonel Bruce Vaughan undertook the task of rebuilding this part of the Hospital, and in response to his appeal £8,000 was subscribed in less than a year, and the whole department has been rebuilt and is said to be one of the most efficient in the provinces. Again, in 1907, he urged the necessity of increasing the accommodation to meet the demand of the ever-increasing number of patients waiting admission, and in the short space of nine months the £30,000 asked for and required for the addition of the new wing was obtained. It will, when completed, add 78 beds, making the total accommodation 272, and it rests with the public to complete, by providing the funds for its maintenance, "one of the finest efforts of hospital work accomplished in the United Kingdom within recent years."

### LONDON HOMŒOPATHIC HOSPITAL.

The past year has been most eventful for this hospital. It has witnessed the inauguration of the long-looked-for extension which has become necessary if the institution is to continue to adequately perform the work that falls upon it. The new Sir Henry Tyler wing, as the extension is named, will add largely to the in- and out-patient accommodation, and it is estimated that when in operation the in-patients will increase from 1,100 to 2,000 per annum, and the out-patients will grow to 20,000 The cost of the new wing is to be £21,000, and the extension of the site £11,000, totalling some £32,000, which has been subscribed, and £2,500 out of the £3,500 that will be needed to furnish the new ward has been contributed. What the board now has to do is to endeavour to raise some £6,000 required for extensive alterations to be made in the interior of the old hospital building (built 16 years ago) to bring it up to present-day requirements. The operating theatre is to be enlarged, and new anæsthetising and sterilising rooms are to be formed. The fourth and fifth floors in the centre are replanned and enlarged. The kitchen department is to be re-arranged and much increased, and many minor changes are made elsewhere. The London Homœopathic Hospital may fairly rank as one of London's general hospitals, seeing that its doors are open day and night for the reception of cases of accident and emergency. Donations or annual subscriptions will be welcomed by Mr. Edward A. Attwood, the Secretary, at the London Homœopathic Hospital, Great Ormond Street, W.

